# Fresh Mushroom Preservation Techniques

**DOI:** 10.3390/foods10092126

**Published:** 2021-09-09

**Authors:** Katy Castellanos-Reyes, Ricardo Villalobos-Carvajal, Tatiana Beldarrain-Iznaga

**Affiliations:** 1 Facultad de Ciencias Tecnológicas, Universidad Nacional de Agricultura, Carretera a Dulce Nombre de Culmí, km 215, Barrio El Espino, Catacamas 16201, Honduras; kcastellanos@unag.edu.hn; 2 Food Engineering Department, Universidad del Bío-Bío, Av. Andrés Bello 720, P.O. Box 447, Chillán 3780000, Chile; tatybeldarrain@gmail.com

**Keywords:** fresh mushrooms, nutraceutical value, preservation, sustainable packaging

## Abstract

The production and consumption of fresh mushrooms has experienced a significant increase in recent decades. This trend has been driven mainly by their nutritional value and by the presence of bioactive and nutraceutical components that are associated with health benefits, which has led some to consider them a functional food. Mushrooms represent an attractive food for vegetarian and vegan consumers due to their high contents of high-biological-value proteins and vitamin D. However, due to their high respiratory rate, high water content, and lack of a cuticular structure, mushrooms rapidly lose quality and have a short shelf life after harvest, which limits their commercialization in the fresh state. Several traditional preservation methods are used to maintain their quality and extend their shelf life. This article reviews some preservation methods that are commonly used to preserve fresh mushrooms and promising new preservation techniques, highlighting the use of new packaging systems and regulations aimed at the development of more sustainable packaging.

## 1. Introduction

Mushrooms have become an essential part of the human diet, having been used in food and medicine for several centuries. These compounds have attractive flavor, aroma, and nutritional value due to such components as high-quality proteins, vitamins, minerals, unsaturated fatty acids, and dietary fibers and their low caloric content [1]. In recent years, their production and consumption worldwide have experienced a significant increase. According to the FAOSTAT database, world production of mushrooms and truffles has increased from 7.5 million tons in 2009 to 11.8 million tons in 2019 [2]. Due to the recognition of their beneficial effects on human health derived from their bioactive compounds and nutraceutical compounds, mushrooms are considered a functional food and a source of nutraceuticals [3]. They are also an attractive food for vegetarians because they contain essential amino acids found in animal proteins [4] and are considered the only natural source of vitamin D_2_ for vegetarians and vegans. For these reasons, consumers have been increasingly incorporating them into their diet, stimulating a notable increase in their production and consumption [3]. Per capita consumption of mushrooms has increased from 1 kg in 1997 to around 4 kg in 2012 [5].

Mushrooms are highly perishable due to the absence of a protective cuticle, their high respiratory rate, and their high moisture content. As a result, they are exposed to mechanical damage, microbial attack, weight loss, and enzymatic browning, which generate a rapid loss of quality postharvest. The shelf life of *Agaricus bisporus, Pleurotus ostreatus, Lentinula edodes* mushrooms can reach one to three days at ambient temperature (20–25 °C) [6,7,8] and five to seven days under refrigeration (4 °C) for *Agaricus bisporus, Pleurotus ostreatus* mushrooms [6,9]. However, *Lentinula edodes* mushrooms have three to five days (4 °C) of post-harvest shelf life [7]. This greater perishability is associated with its higher respiratory rate compared to other mushrooms [10].

To maintain the bioactive compounds and/or secondary metabolites responsible for these healthy properties, appropriate preservation techniques are required to maintain the quality and extend the shelf life of fresh mushrooms [11].

This review aims first to review the production and main species of commercialized mushrooms and their currently growing demand. Highlighting their chemical and nutritional composition and the nutraceutical and therapeutic properties exerted by some bioactive compounds on the health of the consumer. Factors associated with the nature of mushrooms and factors related to storage conditions, which can affect the quality of fresh mushrooms, are also reviewed. Due to the need to guarantee the availability of bioactive and nutraceutical compounds and maintain the postharvest quality of mushrooms, different methods of preservation of fresh mushrooms are also reviewed—some traditional, such as cooling, washing with antimicrobial and antibrowning agents, irradiation, and the use of packaging, and other more recent ones, such as pulsed electric fields, ultrasound, and plasma. Finally, the review covers the use of new packaging in the preservation of the quality of fresh mushrooms. Specifically, the use of modified atmospheres, development of microperforated packaging and the use of humidity regulators. Additionally, the latest trends in food packaging, such as active and smart packaging, for the conservation of fresh mushrooms are described. In the last part, some considerations related to the development of more sustainable packaging under the concept of a circular economy are also reviewed [12], as is the existence of some laws and regulations aimed at reducing waste generation, promoting reuse and recycling, and recovering recyclable materials.

## 2. Characteristics of Mushrooms

Mushrooms are the fleshy fruiting and spore-bearing body of a fungus; they are produced on the soil or in its substrate, mainly by the *Basidiomycota* and *Ascomycota* groups [13]. In Asia, for centuries mushrooms have been used as a food source and for medicinal purposes due to their flavor, nutritional components, and biologically active components [14]. There are more than 14,000 species of mushrooms, but approximately 2000 are edible. Approximately 35 species of mushrooms are cultivated commercially, while approximately 200 wild species are used for medicinal purposes [15]. The market for edible mushrooms is segmented into two types: cultivated mushrooms, which can be available throughout the year thanks to their reproduction under controlled conditions, and wild mushrooms, which are characterized by limited availability that depends on the geographical location and seasonality of their production, which is associated with environmental conditions [16].

The main species of commercialized mushrooms are *Agaricus bisporus*, shiitake (*Lentinus edodes*), and oyster (*Pleurotus spp*.), which represented almost 76% of the global market share in 2013 [17]. According to the Food and Agriculture Organization of the United Nations, approximately 10.2 million tons of mushrooms were produced worldwide in 2017. Between 2007 and 2017, Asia was responsible for 76.0% of the world production, followed by Europe (17.2%), America (5.9%), Oceania (0.6%), and Africa (0.2%) [2].

Traditionally, mushrooms were consumed as food due to their high nutritional value and culinary characteristics, some species of mushrooms being appreciated for their high value in gourmet cuisine [18]. Recent studies have documented that mushrooms have health benefits [1] derived from their nutraceutical compounds and bioactive compounds, leading some to consider them a functional food and source of nutraceuticals [4]. These would be some possible explanations for the growing consumption of mushrooms in recent decades.

### 2.1. Chemical Composition and Nutritional Value

For thousands of years, mushrooms have been used as a food source due to their chemical composition, which makes them attractive from a nutritional point of view [19]. Today they are in high demand partly because they are low in calories, carbohydrates, fat, and sodium and are free of cholesterol [20].

The chemical composition and nutritional value of mushrooms can be affected by the growth stage and postharvest conditions and can vary greatly between species [16]. According to the review carried out by Kalac, the median values of the proximate composition of some cultivated mushroom species are 208, 22, 70, and 682 mg kg^−1^ dry matter of crude protein, lipids, ash, and carbohydrate, respectively, and 419 kcal kg^−1^ fresh matter. The dry matter content of mushrooms is very low, at approximately 80–140 g kg^−1^, and is mainly due to the high-water content of freshly cultivated mushrooms. This last condition is one of the main factors responsible for the short shelf life of fresh mushrooms [16].

From a nutritional point of view, mushrooms are considered a valuable source of protein due to their high content (15 to 35% of dry weight), reflecting the belief that mushrooms are an effective substitute for meat, and their nutritional value can be compared with that of many plant species [21]. Although they have lower protein concentrations than animal meat, they have more than other foods, including dairy products [4]. Mushroom proteins have a higher nutritional value than most vegetable proteins [11] because they contain all essential amino acids [22]. Mushrooms are also considered an attractive food for vegetarians because they contain essential amino acids found in animal proteins [23].

### 2.2. Nutraceutical and Therapeutic Properties

Mushrooms have become increasingly important in the diet since they are considered functional foods due to their possible health benefits [24]. They contain bioactive nutraceutical components such as polysaccharides (β-glucans), dietary fibers, glycoproteins, unsaturated fatty acids, phenolic compounds, tocopherols, ergosterols, and lectins [25]. Some reports explain its antitumor action by their stimulation of the immune system through the action of their polysaccharides [13,26] and immunomodulatory action [27], for which they have been used in complementary and alternative medicine as a drug against cancer [28]. Mushrooms are also considered to help prevent atherosclerosis because they are rich in derivatives of ergosterol, eritadenine, β-glucans, and HMG-CoA reductase inhibitors [29].

Mushrooms are the only nonanimal foods that contain vitamin D, so they represent the only vegetarian and vegan source of vitamin D_2_ [30]. The exposure of mushrooms to artificial UV light or sunlight stimulates the conversion of ergosterol into vitamin D_2_ through a series of thermal and photochemical reactions, in greater quantity than the daily vitamin D requirements of humans [31,32]. Considering the restrictions on the mobility of people imposed by the current COVID-19 pandemic, which can reduce their sun exposure and therefore their synthesis of vitamin D, the consumption of mushrooms appears to be an excellent dietary source of vitamin D that can cover the recommended daily intake.

Mushrooms are considered an excellent option for the treatment of hypertension because they contain active antihypertensives such as peptides D-mannitol, D-glucose, D-galactose, D-mannose, triterpenes, and potassium [33]. In addition, their positive effects on neurological health, synaptic and neuroregenerative plasticity, neurite growth, and inhibition of acetylcholinesterase and β-secretase have been studied [34,35,36], which are crucially involved in the pathophysiology of Alzheimer’s disease, dementia, depressive disorder, and cognitive decline [37,38].

Mushrooms have also been reported to have antiviral, antibacterial, antidiabetic [39], antioxidant [20,40], anti-inflammatory properties and to help prevent the risk of stroke and certain types of cancer [1]. These properties have been attributed to the presence of secondary metabolites present in mushrooms, such as phenolic compounds, and their antioxidant ability [41].

Due to the various beneficial effects of mushrooms on human health, consumers have been increasingly incorporating them into their diet, generating a notable increase in their production and consumption [3]. To maintain the bioactive compounds and/or secondary metabolites responsible for these properties, adequate preservation techniques are required to maintain the quality and extend the shelf life of fresh mushrooms [11].

## 3. Factors Affecting the Quality of Fresh Mushrooms

Fresh mushrooms are one of the most perishable food products, and after harvest, they experience a rapid degradation of their quality. The loss of moisture, color changes, textural changes, microbial deterioration, and loss of nutrients and flavor are the parameters that most frequently affect their quality [42], limiting their shelf life to only one to three days at room temperature [9] or five to seven days when stored under refrigeration [5]. Mushrooms are highly perishable products because they do not have a cuticle layer to protect them against mechanical damage, water loss, or microbial attack. In addition, the high moisture content (85–95%) of fresh mushrooms [43] and their high respiration rate (200–500 mg kg^−1^ h^−1^ at 20 ± 1 °C for *Agaricus bisporus* and 640.8 mg kg^−1^ h^−1^ at 10 °C for *Lentinus edodes*) [44,45] contribute to rapid senescence by promoting microbial attack and enzymatic browning [46].

Due to their high moisture content and respiratory activity, mushrooms experience a rapid loss of moisture after harvest, resulting in a continuous loss of weight, shrinkage, and loss of turgor, accelerating the degradation of their quality [47]. A weight loss of 5–10% of its fresh weight causes wilting of the mushroom, making it unsuitable for commercial sale [48]. Due to the loss of water, the firmness of the mushrooms *Agaricus bisporus* stored for 16 days at 4 °C experiences a decrease in firmness from 17.32 N to approximately 13 N [49]. Similarly, mushrooms *Lentinus edodes* stored under the same conditions show a decrease in firmness from 3.4 N to 2.1 N [50] while *Pleurotus ostreatus* experienced an important decrease in firmness from 162.8 to 1.49 N/m^2^ after 15 days of storage at 4 °C [51].

The color of mushrooms, particularly in white strains, is one of the quality attributes of greatest commercial importance because consumers use it to decide whether to purchase [52]. The surface of mushrooms is prone to browning due to microbial contamination or enzymatic activity [52] and due to mechanical injuries that occur during handling and transport [53]. Tyrosinase, an enzyme belonging to the polyphenol oxidase family, is one of the main enzymes responsible for the browning of *Agaricus bisporus* and *Pleurotus ostreatus* due to its high content [51,54]. The browning of *Lentinus edodes* also is ascribed to the action of polyphenylene oxide enzyme and microorganisms on its tissue [55]. On the other hand, during storage in refrigeration (4 °C and 85% relative humidity), the antioxidant compounds of mushrooms (total phenolics) decrease, and the lipid peroxidation process of the membrane increases [56].

Some previous reviews have identified a series of factors that can affect the quality attributes of mushrooms after harvest [57], classifying them into internal factors related to the mushroom itself (water activity, respiration rate, and microbial activity) and external factors related to storage (storage temperature, relative humidity, and mechanical damage).

Water activity (a_w_) is an important factor that influences the quality of mushrooms. Their high content of free water has been related to lipid oxidation, microbial stability, enzymatic and nonenzymatic activity, and changes in the texture of mushrooms [58].

Respiration is a good indicator of the physiological aging of fresh mushrooms and is affected by temperature and storage time [59]. An increased respiration rate during storage has been linked to decreased weight [60] and the browning of the mushrooms. In the latter case, high humidity and a high surface temperature accelerate the discoloration process [61].

The use of composting in the cultivation of mushrooms can permit a high initial microbial contamination, with microbial counts reaching 5.2 to 12.4 log colony-forming units (CFU)/g [62]. In turn, a high microbial load can significantly reduce the quality of mushrooms, generating browning and a stained appearance and deterioration [57]. *Pseudomonas* spp. and *Flavobacterium* spp. are the two groups of bacteria that predominate in stored mushrooms, *Pseudomonas tolaasii*, *Pseudomonas fluorescens* being the microorganism causing the bacterial staining in cultivated *Agaricus bisporus*, *Pleurotus eryngii, Pleurotus ostreatus* and *Lentinula edodes* [63,64]. *Penicillium* species has also been one of most frequently isolated from the ambient air of *Pleurotus ostreatus* cultivation facilities [65]. Occurrences of pathogenic bacteria such as *Campylobacter jejuni*, *Staphylococcus aureus*, *L. monocytogenes*, *Ewingella Americana* in commercial fresh mushroom have also been reported [63,66,67,68] and *L. monocytogenes* has been isolated in some wild mushroom species [69].

Among the external factors, the storage temperature is the one that can most affect the postharvest quality of the mushrooms because most of the physical, biochemical, and microbiological deterioration reactions are temperature dependent. A higher storage temperature accelerates the senescence, browning, weight loss, and textural loss of mushrooms [70]. Storage of mushrooms for one day at room temperature (20–25 °C) produces an opening of the cap, browning, elongation of the stem, and loss of turgor [71]. Likewise, when the storage temperature increases from 2 to 18 °C, holding the relative humidity at 86%, the transpiration rate of fresh mushrooms increases from 1.82 to 3.88 g/kg [72].

Relative humidity is another factor that influences the quality of the mushroom after harvest. Low relative humidity can produce an excessive loss of water, which causes a reduction in turgor, pore closure, and increased enzymatic activity, finally triggering rapid cellular disaggregation [57]. A weight loss greater than 5% of fresh mushrooms weight produces an undesirable deterioration in its quality [47]. In contrast, a very high relative humidity can favor the condensation of water on the surface of the mushroom, generating conditions for the growth of microorganisms that eventually cause its decomposition [73]. Considering that fresh mushrooms are usually packed in plastic trays and over-wrapped with a film and stored under refrigeration temperature to prevent moisture loss [61], it is necessary to design a packaging system that provides an adequate moisture balance inside the trays to avoid moisture loss or moisture condensation inside the trays [6]. The effect of different relative humidity levels on the quality of fresh mushrooms (*Agaricus bisporus*) was investigated by Cliffe-Byrnes, finding that a relative humidity of 96% inside the packaging was the optimal level to maintain the quality of the mushrooms [74].

A weight loss of 8.35% was observed in oyster mushrooms when they were stored for five days in saturated steam conditions at 2 °C [72], while for shiitake mushrooms a weight loss of 5.34% was obtained after 18 days of storage at 4 °C and a relative humidity between 85 to 90% [75], similarly for *A. bisporus* mushrooms, a weight loss of 4.3% was obtained when stored for 16 days at 4 °C and a relative humidity of 80 to 90% [76].

## 4. Conservation Methods for Fresh Mushrooms

Based on the above discussion on the beneficial health effects of some bioactive compounds and/or secondary metabolites present in mushrooms and considering all the factors that can affect the attributes of their quality, the need to apply conservation methods that effectively reduce the deterioration of quality, lengthen the shelf life, and retain the nutritional value of fresh mushrooms is evident.

The methods that have usually been used to extend the shelf life of mushrooms are cooling and packaging in plastic trays coated with a perforated polyvinyl chloride (PVC) film [61]. However, there are other techniques that can be used for this same purpose and that can be used in a complementary way to achieve more efficient results.

**Figure 1:**
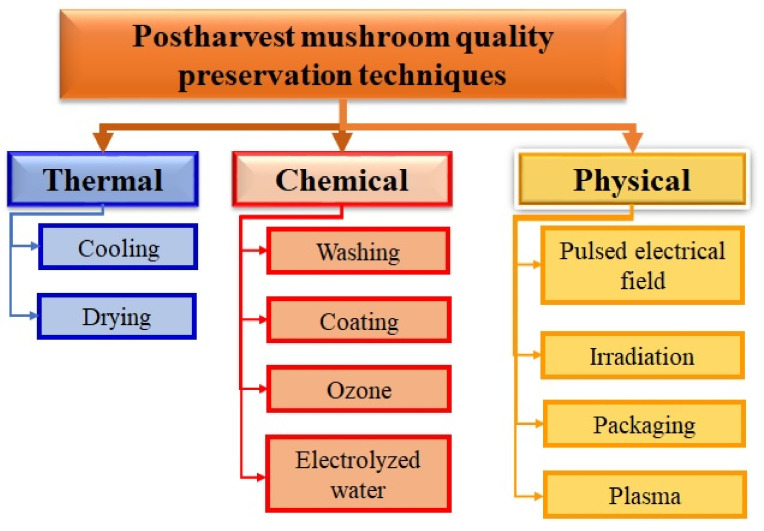
Mushroom quality preservation techniques.

Recently, Zhang et al. [57] reviewed postharvest preservation techniques used to maintain the quality and extend the shelf life of mushrooms. These authors classified these preservation techniques into three categories: thermal, physical, and chemical [57]. Figure 1 shows a summary of the strategies that have been studied to slow or halt chemical deterioration and microbial growth and avoid recontamination of the mushroom.

### 4.1. Thermal Processes

#### Cooling

After harvest, the rapid removal of heat from the mushrooms and maintenance of a low storage temperature are vital to extend their shelf life. Low temperature is effective in slowing the growth of microorganisms, reducing the respiration rate of mushrooms, and minimizing their moisture loss [77]. By decreasing the storage temperature from 25 to 3 °C, 75% less browning was obtained in the mushrooms [78]. The effect of refrigerated storage on the nutritional composition and active compounds was studied in *Flammulina velutipes* fruiting body. The results indicated that natural antioxidant 2-thiol-L-histidine-betaine (L-ergothioneine) content decreased significantly after eight days of storage at 5 °C, both in dark and fluorescent light conditions, while the total phenols content in mushrooms stored under fluorescent light increased significantly during 10 days of refrigeration. However, the antioxidant capacity decreased after five and eight days of storage under dark and light conditions [79].

Due to their high moisture content and porous structure, vacuum cooling is the most widespread and commercially used cooling technology to preserve mushrooms [80,81]. The cooling process is produced by the evaporation of moisture from the product. Compared to conventional cooling methods, vacuum cooling can significantly reduce the cooling time as well as the rate of microbial growth. However, the high investment costs and the greater weight loss compared to that seen under conventional cooling usually limit its application [82]. A comparison of vacuum cooling with other conventional cooling methods used in fresh horticultural products has been made by Kader and Rolle [83]. One of the advantages that stand out is the short vacuum cooling time (0.3–2.0 h) compared to forced air cooling (1.0–10.0 h) and cold-storage room of (20–100 h). In terms of energy consumption, vacuum cooling is more efficient, but the capital cost is higher than traditional cooling systems. In turn, vacuum cooling produces a greater loss of moisture, due to the partial removal of moisture during the cooling process. The effect of vacuum cooling and conventional cooling on browning and hyphal structure of *Agaricus bisporus* mushrooms was investigated by Burton et al. [84]. No differences were found in the browning and hyphal structure of the mushrooms when using both cooling methods and subsequent maintenance at 5 °C. However, less browning was observed in the vacuum-cooled mushrooms and subsequent storage at 18 °C compared to those conventionally cooled. On the contrary, the vacuum-cooled mushrooms and stored at 5 °C for 102 h experienced a greater weight loss (1.7%) compared to the conventionally cooled ones. Mittal et al. investigated the effect of four pre-cooling methods (hydrocooling, room cooling, forced air cooling, vacuum cooling) and the use of polybags (polypropylene and polyvinyl chloride) and punnets (high impact polystyrene and polyvinyl chloride) on the shelf life of *Agaricus bisporus* stored under environmental conditions (14–16 °C, relative humidity 56–83%), in terms of its physicochemical quality and sensory evaluation. They found that vacuum cooling was the best pre-cooling method followed by forced air cooling and room cooling and the best packaging materials were punnet packages (HIPS and PVC) followed by PP and PVC, extending the shelf life and maintaining the quality of mushrooms up to 4 days [85]. In turn, Tao et al. investigated the effect of vacuum cooling treatment and different storage conditions (cold room and modified atmosphere packaging) on the antioxidant enzyme activity of *Agaricus bisporus* mushrooms. Vacuum-cooled mushrooms showed increased superoxide dismutase, catalase, peroxidase and polyphenoloxidase activity. In contrast, malondialdehyde levels and superoxide anion generation slightly decreased. During the storage, the highest expression of the enzymatic antioxidant system was found in the mushroom stored under modified atmosphere packaging with vacuum cooling treatment [86].

### 4.2. Chemical Treatments

#### 4.2.1. Washing with Antimicrobial and Antibrowning Agents

The use of compost in the cultivation of mushrooms can produce a high initial contamination, so it is necessary to remove adhered dirt and microorganisms from the surface of the mushrooms through a washing or rinsing process to inhibit microbial deterioration [77]. However, because washing raises the moisture content of mushrooms, this makes them more prone to the development of microorganisms and browning. For this reason, the addition of antimicrobial and antibrowning agents to wash water is common [57]. Sodium metabisulfite was used in the washing of mushrooms, producing excellent results to maintain the initial whiteness, but it was not effective in inhibiting the growth of decomposing bacteria [87]. Due to severe allergic reactions in asthmatic consumers, the United States Food and Drug Administration (FDA) prohibited its use in fresh mushrooms in 1986 [57], and it has been replaced by other agents, such as chlorine dioxide, citric acid, EDTA, hydrogen peroxide (H_2_O_2_), among others [87].

The effect of different washing solutions that had H_2_O_2_ (1.5%, 2.5%, and 3.5%), citric acid (0.5%, 1.5%, and 2.5%), or EDTA (2%, 4%, and 6%), applied to fresh whole mushrooms (*A. bisorus*) for 10 min followed by refrigerated storage for 12 days, was studied by Gupta and Bhat [88]. Their results indicated that 2.5% citric acid was the most effective solution at preserving the postharvest quality of the mushrooms. Acid citric was the most effective in controlling weight loss, maturity index and microbial growth, but induced a slight yellowness on mushroom surface. Combined treatments have also been studied, such as by Guan et al. [89]. They studied the application of water and H_2_O_2_ before irradiation of mushrooms with UV-C light (254 nm, 0.45 kJ m^−2^), which were then stored for 14 days at 4 °C. Washing with water followed by UV-C treatment yielded a reduction of 0.77 log CFU/g in *E. coli* O157:H7. On the other hand, when washing with 3% H_2_O_2_ water before the application of UV-C light, a greater microbial reduction was achieved (0.85 log CFU/g) and an increase of total phenolic and ascorbic acid contents of mushrooms during 14 days of storage. This showed the best performance in the inhibition of lesions and browning in mushrooms. The impact of wash treatments on the nutritional value and structure of mushroom *Agaricus bisporus* was investigated by Sapers et al. [90]. They reported a minimal effect of a H_2_O_2_ wash solution and subsequent application of sodium erythorbate (browning inhibitor) on the content of carbohydrate, protein, fat, ash, vitamin, and phenolic compounds. Minimal hyphal damage was also observed with these wash solutions. Only a 19% decrease in free amino acids was observed due to a leaching phenomenon during washing.

#### 4.2.2. Coatings

The use of edible coatings on fruits and vegetables has been shown to extend their shelf life during storage [91]. The coating acts as a semipermeable barrier to O_2_, CO_2_, and moisture, modifying the gaseous composition at the coating–product interface [92] Thus, they can help reduce respiration, delay ripening, reduce moisture loss, and maintain the firmness and color of mushrooms [93]. Recently, interest in the use of coatings has increased because coatings can serve as a vehicle to incorporate a wide range of food additives, including colorants, flavors, nutrients, species, antioxidants, and antimicrobial agents, which can extend shelf life and reduce the risk of pathogen development on the surfaces of foods [94,95].

Coatings with various biopolymers have had beneficial effects on the preservation of mushrooms. The use of gum arabic enriched with natamycin as an edible coating on shiitake mushrooms maintained firmness, delayed changes in the soluble solids, total sugar and ascorbic acid, sensory quality and reduced the yeasts and mold counts, extending their shelf life by 16 days [96]. Similar results were obtained when applying alginate coatings on button mushrooms and storing them in an atmosphere of 100% O_2_. The mushrooms achieved a high level of firmness and delayed browning, cap opening, and changes in soluble solids, total sugars, and ascorbic acid, achieving a shelf life of 16 days [9]. The combined use of aloe vera and gum tragacanth as a coating on button mushrooms minimized weight loss and browning and decreased texture loss during storage for 13 days at 4 °C. According to Jiang et al. [97], treatment with a chitosan–glucose complex coating maintained the firmness of the tissue, reduced the microbial counts, significantly reduced the loss of ascorbic acid and inhibited the increase in the respiratory rate in shiitake mushrooms during 16 days of storage at 4 °C. The combination of gum tragacanth and *Zataria multiflora Boiss* essential oil and *Satureja khuzistanica* essential oil managed to maintain 93.47% and 92.4% of the firmness of the tissue of the mushrooms, respectively, reduced the microbial counts, and decreased the browning index. Regarding the effect of these coatings on the content of active compounds and the nutritional composition of the mushrooms, each of these coatings was able to retain 33.3% and 32.9% of total phenolic content and 31.9% and 30.9% of ascorbic acid, compared to uncoated mushrooms [95,98]. In addition, Zhu et al., finding that edible coating prepared using sodium alginate, enriched with 1% (*v*/*v*) thyme essential oil, 0.3 g/L L-cysteine, and 0.4 g/L nisin, increased the postharvest quality of *Pholiota nameko* at 4 °C. This edible coating significantly inhibited the weight loss, degree of browning, malondialdehyde content, polyphenol oxygenase, peroxidase, and cellulase activity of *P. nameko*. In addition, edible coatings preserved the soluble sugar, ascorbic acid, and soluble protein contents [99]. Recently, Liu et al. investigated the effect of coating shiitake mushrooms with a polysaccharide isolated from *Oudemansiella radicata* on their quality and flavor after 18 days of storage at 4 °C. The coated mushrooms had a significant improvement in quality, including reduced weight loss, improved firmness, reduced browning, decreased malondialdehyde content, and improved microstructure. They also had a higher concentration of superoxide dismutase and catalase, as well as a higher content of certain types of monosodium glutamate-resembling amino acids, umami 5′ -nucleotides and 1-octen-3-ol. The coated mushrooms had a higher nutritional content compared to the control. The coatings delayed the breakdown of proteins and carbohydrates and retained a higher content of ascorbic acid and phenolic compounds, preserving their antioxidant capacity [75].

#### 4.2.3. Ozone

Ozone is a powerful antimicrobial agent used to extend the shelf life of food. Due to its strong oxidizing ability, ozone produces rapid microbial inactivation by reacting with intercellular enzymes and cellular components. Considering that after the decontamination process, the excess ozone rapidly decomposes into oxygen, the USFDA considers it a sanitizing agent that can be in direct contact with food [100]. Along with these beneficial effects, the use of ozone as a disinfectant can also have some drawbacks, such as its high instability in the gaseous state, rapid decomposition that can cause recontamination, corrosion at high concentrations, and high investment cost [100]. The effect of ozone on the microbiota and pathogens in button mushrooms was studied by Akata et al. [101]. They found that exposure for 60 min to ozone gas at concentrations of 2.8 and 5.3 mg/L caused logarithmic reductions of 2.44 and 3.07 in the aerobic plate counts and logarithmic reductions of 3.61, 2.80, and 3.41 in the *Salmonella*, *L. monocytogenes*, and *E. coli* O157:H7 counts, respectively. Watanabe et al. found that the effects of ozone on the chemical composition of the mushroom *Pleurotus ostreatus* were an increase in weight, water content, proteins, Ca, K, Zn, riboflavin, and ascorbic acid and a decrease in carbohydrates, iron, and thiamine [102].

Oyster mushrooms pre-treated with 10 and 15 ppm of ozone for 5 and 10 min and packed in HDPE containers were investigated by Anjaly et al. [103]. Their results indicated that the ozone pre-treatments combined with the packaging condition was effective in extending the shelf life of the mushrooms up to 10 days. A 15 ppm ozone treatment for 10 min achieved a 2.25 log CFU reduction from aerobic plate counts. Even though the application of ozone generated an initial increase in the browning of the mushrooms compared to the control, during storage for 10 days the browning reaction was controlled by ozone. The loss of weight and texture of the mushrooms was not affected by the ozone treatments. In other work, the effect of the application of different doses of gaseous ozone (0.05, 1.0 and 2.0 mg/L) and ozonation time (30 and 60 min) on the physicochemical properties of *Agaricus bisporus* stored at 2 °C for 14 days was investigated by Zalewska et al. [104]. The total phenolic content and the total antioxidant activity of the mushrooms decreased while the weight loss increased with the application of ozone. In turn, the external browning process during storage was accelerated with the application of ozone, while a delay in internal browning was observed. Liu et al. found that the intermittent application of ozone at a concentration of 3.21 mg/m^3^ was effective in maintaining the quality and prolonging the shelf life of Shiitake mushrooms stored at 4 °C and 85–95% relative humidity [105]. Ozone treatment inhibited the activity of polyphenol oxidase and increased the activities of phenylalanine ammonium-lyase, peroxidase, superoxidase dismutase and catalase, effectively delaying the browning of the mushroom. In turn, high levels of total phenols, soluble protein content were maintained, and a reduced accumulation of free amino acids was produced.

#### 4.2.4. Electrolyzed Water

Electrolyzed water is a promising broad-spectrum disinfectant that can be used in the food industry [106]. It is generated by electrolysis of a saline solution, and its antimicrobial activity is determined by the concentration of free available chlorine that forms hypochlorous acid (HClO), its oxidation–reduction potential (ORP), and the combined effect of these [107].

The combined use of electrolyzed water (5, 25, 50 and 100 mg/L) and a passive modified atmosphere in button mushrooms was researched by Aday et al. [108]. The results indicated that the mushrooms treated with 25 mg/L presented a lower browning index than the untreated mushrooms and had a delayed loss of weight and texture. In another study, bactericidal effects were compared between low-concentration electrolyzed water and four other disinfectants (water electrolyzed with strong acid, 1% citric acid, aqueous ozone, and sodium hypochlorite solution). Low-concentration electrolyzed water presented greater antimicrobial efficacy than the other disinfectants, achieving reductions of 1.35, 1.08, and 1.90–2.16 log CFU/g in total aerobic bacteria, mold and yeasts, and foodborne pathogens, respectively, after a 3 min treatment at room temperature (23 ± 2 °C) [109].

### 4.3. Physical Treatments

#### 4.3.1. Pulsed Light

Pulsed light (PL) is a nonthermal technology used for rapid inactivation of pathogenic microorganisms to prevent food spoilage. It has been approved as a decontamination technology by the FDA [110]. This technique consists of applying short-duration, intense pulses of a broad spectrum of light onto the surface of the food for microbial inactivation [111]. The main mechanism of inactivation of PL corresponds to the photochemical effect produced by the broad UV spectrum and the density of the energy used, which produces structural changes in the DNA of bacteria, viruses, and other pathogens, which prevents their reproduction [112]. The application of high pulsed light fluencies (12 and 28 J cm^−2^) affected the texture of sliced mushrooms (*Agaricus bisporus*) due to thermal damage induced by the treatments. In turn, greater browning was promoted due to higher polyphenol oxidase activity and the content of phenolic compounds, vitamin C and antioxidant capacity were significantly reduced. However, the application of PL at low doses (4.8 J·cm^−2^) could extend the microbiological shelf life of fresh button mushrooms in slices by 2–3 days without dramatically affecting the texture or antioxidant properties [113].

#### 4.3.2. Ultrasound

Ultrasound is mechanically vibrating waves with a frequency greater than 20 kHz. It is classified as low-intensity ultrasound (<1 W/cm^2^, frequencies of 0.1–20 MHz) and high-intensity ultrasound (10–1000 W/cm^2^, frequency less than 0.1 MHz). High-intensity ultrasound is used as an antimicrobial treatment in food [114]. The antimicrobial effect is related to the phenomenon of cavitation produced in the gas bubbles, which generates a destructive effect on the cells of the microorganisms present in the sonicated medium [115]. Low-concentration electrolyzed water combined with ultrasound managed to better maintain the surface color and firmness of fresh sliced button mushrooms compared to the treatment with only electrolyzed water [114].

#### 4.3.3. Irradiation

Gamma irradiation of several products has been approved as safe by the FDA. A dose of up to 1 kGy has proven useful to maintain the quality and extend the shelf life of edible wild mushrooms, producing a decrease in enzymatic browning due to a delay in the activity of polyphenol oxidase [116,117]. In another study, the effects of different irradiation doses (0.5, 1, 3.1, and 5.2 kGy) on the quality of button mushroom slices were studied [118]. Doses above 0.5 kGy significantly reduced microbiological counts to undetectable levels and prevented microbiologically induced browning. Irradiation at 1 kGy was the most effective at extending the shelf life of the mushroom slices. However, the use of gamma irradiation could cause variations in the chemical composition [119]. Gamma irradiation has been investigated in wild mushrooms such as *Lactarius deliciosus*, *Boletus edulis* and *Hydnum repandum* [120,121], evaluating its effect on nutritional composition and antioxidant activity. The results indicated that doses up to 1 kGy allow to maintain the quality and extend the shelf life of mushrooms, without significantly affecting the macronutrients, energetic value, tocopherols and antioxidant activity [120,121].

Ultraviolet radiation is generally classified into three groups: UV-C (200–280 nm), UV-B (280–320 nm), and UV-A (320–400 nm). UV-C irradiation has been authorized by the FDA for use as a disinfectant for the surface treatment of food [92]. The application of UV-C irradiation (4 kJ/m^2^) on shiitake mushrooms and storage in a modified atmosphere for 15 days at 1 °C and 95% relative humidity resulted in a high maintenance of firmness, higher contents of flavonoids and ascorbic acid, and greater antioxidant ability of mushrooms [122].]. In another study, the effect of UV-C (0.45–3.15 kJ/m^2^) on the microbial load of fresh button mushrooms stored for 21 days at 4 °C was studied. A reduction of 0.46–1.13 CFU/g and 0.63–0.89 CFU/g was achieved in the *E. coli* O157:H7 count and the total aerobic bacterial counts [67]. Mushrooms are rich in sterols, mainly ergosterol. When they are exposed to UV radiation, the conversion of ergosterol into vitamin D_2_ occurs through a photolysis process [123]. Taofiq et al. have reviewed numerous studies evaluating the effect of different sources of UV radiation such as Ultraviolet A, Ultraviolet B, Ultraviolet C, on the production of vitamin D_2_ in various species of fresh cultivated mushrooms (*Agaricus bisporus*, *Lentinula edodes*, *Pleurotus ostreatus*) and also in other species of mushrooms (*Agaricus bitorquis*, *Agrocybe cylindracea*, *Auricularia polytricha*, *Boletus edulis*, *Cantharellus tubaeformis*, *Cordyceps militaris*, *Flammulina velutipes*, *Hericium erinaceus*, *Hypsizygus marmoreus*, *Lentinus squarrosulus*, *Lentinus polychrous*, *Pholiota nameko*, *Pleurotus citrinopileatus*, *Pleurotus cystidiosus*, *Pleurotus djamor*, *Pleurotus eryngii* var. ferulae, *Pleurotus pulmonarius*, and *Volvariella volvacea*. These authors point out that these mushrooms present a considerable amount of vitamin D_2_ after exposure to UV radiation [31]. Other authors investigated the effect of UV-B on the concentration of vitamin D_2_ in sliced Shiitake mushrooms (*Lentinus edodes*) and button mushrooms (*Agaricus bisporus*). The vitamin D_2_ concentration, in both types of mushrooms, increased with the increase of the irradiation dose. They also found that the irradiation on sliced mushrooms was more efficient in increasing the content of vitamin D_2_ compared to the whole mushroom, due to its greater exposure area and that UV irradiation acts only on the surface of the mushrooms [124]. Another investigation evaluated the application of UV-B light irradiation for 2 h on the content of vitamin D_2_ in the edible fruit bodies and mycelia of 11 species of fresh mushrooms. After irradiation, the content of vitamin D_2_ in the fruit bodies of the mushrooms increased significantly from 0–3.93 to 15.05–208.65 µg/g and in the irradiated mycelia of golden oyster, oyster and pink oyster mushrooms increased from 0.28–5.93 to 66.03–81.71 µg/g. UV-B irradiation had a slight effect on the content of ergothioneine (0.71–3.13, 0.63–0.66 mg/g), flavonoids (5.31–7.26, 1.78–8.21 mg/g) and total phenols (7.15–18.25, 6.36–13.08 mg/g) of fruiting bodies and mycelia respectively, but these authors note that the irradiated samples still contain sufficient amounts of these antioxidant compounds [125].

#### 4.3.4. Plasma

Cold plasma is considered an emerging technology that can be a potential alternative to conventional preservation techniques [126]. Plasma is an ionized gas that is produced by the application of an electric or electromagnetic field to a gas (air, oxygen, nitrogen, argon, helium), making the electrons collide with the molecules or atoms of the gas and producing ionization. Therefore, plasma is composed of chemically active compounds, such as reactive oxygen species, free radicals, superoxide, and others [127]. All these compounds can inactivate a wide range of microorganisms and enzymes without affecting their nutritional, physical, or sensory qualities [128]. Like gaseous cold plasma, plasma-activated water can also inactivate certain microorganisms [129]. The application of gaseous plasma or plasma-activated water in shiitake mushrooms stored for 7 days at 4 °C has been studied by Gavahian et al. [130]. These authors found that either treatment could reduce the microbial counts and color changes of the mushrooms. However, mushrooms treated with plasma-activated water retained better maintain firmness. In another study, the effect of plasma-activated water on the postharvest quality of button mushrooms stored for 7 days at 20 °C was studied [60]. The bacterial and mold counts fell by 1.5 log and 0.5 log after 7 days of storage, the decrease in firmness was delayed and no significant changes were observed in color, pH and antioxidant properties.

#### 4.3.5. High Hydrostatic Pressure (HHP)

High hydrostatic pressure is one of the most used non-thermal technologies in food preservation and processing [131]. HHP is used to improve food safety and the shelf life of plant-based foods thanks to its ability to inactivate enzymes and microorganisms [132,133], maintaining bioactive compounds and with low impact on the nutritional and organoleptic quality [132,134,135]. Solid foods processed at high pressure are subjected to a batch system. In this method the products are packed and sealed before processing, then the food is loaded into a perforated basket that is placed in the pressure vessel. Then the pressure is increased to the set pressure and the product is kept at this pressure for 3 to 10 min, then the pressure is released and the depressurized product is unloaded [136].

The activity of endogenous deteriorating enzymes and enzymes from the growth of microorganisms are associated with color and flavor changes, texture degradation and loss of nutritional value of horticultural products, which considerably reduces their quality and shelf life [137]. The effect of high hydrostatic pressure processing (HPP) at levels of 100–300 MPa and holding time of 3–18 min on the quality characteristics of fresh *Pleurotus eryngii*, such as polyphenol oxidase (PPO), weight loss, color, hardness, and sensory quality were studied [138]. The results showed that with increasing pressure and holding time, the PPO enzyme activity and elasticity of the HPP-processed samples increased after an initial decline, the brightness(L*) value and hardness decreased gradually, while the yellowness(b*) value and rate of weight loss increased. However, during storage (0~12 days) at 4 °C, PPO enzyme activity was lower, rate of weight loss was greater, and extent of changes in color and hardness were smaller in case of HHP-treated samples than those of untreated samples. The optimal processing conditions were 200 MPa and 9 min. The effect of high hydrostatic pressure (HHP) at levels of 300–600 MPa during 2.5–25 min on microbial inactivation and inactivation kinetics in fresh mushroom(*Agaricus bisporus*) was also studied [139]. The results showed that the effects of inactivation were improved with the pressure increasing and prolonging the holding time. The mould yeast and coliforms were more sensitive to HHP than aerobic bacteria, and they were inactivated completely at 400 MPa for 2.5 min. On the other hand, high pressure treatments between 600 and 900 MPa produced less degradation of mushroom texture compared to thermal blanching. In turn, the high pressures increased the permeabilization of cell membranes due to the crystallization of their phospholipids and this higher permeability increased the development of browning [140]. In other study, Lagnika investigated the effects of high-pressure argon (H, 10 MPa at 4 °C for 60 min), ultrasound (U, 20 kHz for 10 min) and their combination treatments (UH) on physico-chemical characteristics of white mushrooms during 9 d of postharvest storage at 4 °C. Mushrooms treated with high pressure argon revealed significant reduction of PPO, mass loss and respiration rate and increased marginally in DPPH activity, total phenolic and flavonoid contents. The application of ultrasound combined with high pressure argon appeared to be the most effective treatment to decrease of PPO [141].

Several works have focused on specifically investigating the inactivation of polyphenyloxidase (PPO) in mushrooms. Gomes et al. reported that a treatment at 600 MPa for 10 min, reduced the activity of mushroom PPO by around 50% and a treatment at 800 MPa for 1 min could not inactivate the mushroom PPO completely [142]. A 28% reduction in mushroom PPO activity was reported by Sun et al. by exposing it to 800 MPa for 10 min at a temperature around 35 °C [143]. Application of ultra-high pressure (UHHP) treatments from 1400 to 1600 MPa for 1 min reduced the activity by 90.4% and 99.2% in the buffer, however a higher enzymatic activity remained in the pure. They suggest that the inactivation of mushroom PPO by UHHP treatment at pressure higher than 1000 MPa is due to the synergistic effect of pressure and the increase in temperature generated by pressurization on the secondary and tertiary structure of mushroom PPO [144].

#### 4.3.6. Packaging

Fresh mushrooms can be packaged in different types of packages, depending on whether they are for wholesale or retail and depending on the transportation requirements and the characteristics of the species. Usually, they are sold in trays or baskets of expanded polystyrene covered with a shrink wrap that can be polyethylene or PVC of different permeabilities and stored in refrigeration [6]. The effects of different packaging techniques on the postharvest conservation of mushrooms are compared in Table 1.

##### Modified-Atmosphere Packaging (MAP)

One technique reported to be effective in extending the shelf life of fresh mushrooms, maintaining their quality, and reducing the loss of nutritious compounds and bioactive nutraceutical components has been modified-atmosphere packaging (MAP) [106]. In this technique, an atmosphere inside the container with a high concentration of CO_2_ and low O_2_ is used, which protects the product against alterations caused by oxidation, attack by microorganisms, and variations in color and aroma [150]. Two types of MAP can be used for the conservation of plant products. In active MAP, the composition of the internal gas in the container is modified by the displacement of the initial gas by an established gas mixture or by the inclusion of a gas-scavenging system within the container. In passive MAP, the gas composition is modified due to the combined effect of product respiration and permeability of the packaging film [151]. However, factors such as the permeability of the packaging materials, composition of the gas mixture, surface area of the sample, and temperature and humidity of storage can affect the effectiveness of MAP for fresh mushrooms [152]. A concentration range of 5–10% O_2_ and 2.5–5% CO_2_ is suggested to preserve white mushrooms [153].

Different studies have reported the beneficial effects of MAP in the conservation of different types of mushrooms. MAP has been used to pack fresh sliced mushrooms (*Agaricus bisporus*) [154], wild mushroom (*Lactarius deliciosus*) [155], and shiitake mushroom (*Lentinus edodes*) [156]. Beit-Halachmy and Mannheim noted that MAP can maintain the quality of *Agaricus bisporus*, but if the mushrooms respire faster than expected or if they are exposed to temperature fluctuations, MAP can produce a negative effect [157]. On the other hand, Li et al. found that the storage of *Pleurotus eryngii* in an atmosphere with high concentration of carbon dioxide and low levels of oxygen (2% O_2_ and 30% CO_2_) at 20–25 °C and a relative humidity of 90–95%, extended the shelf life of the mushrooms significantly compared to the control. This gas mixture maintained the sensory characteristics of the mushrooms, reducing the production of reactive oxygen species (ROS) through increased antioxidant enzyme activities during storage [158].

Most of the commercial packaging materials used in the packaging of fresh products have a very low gas permeability [6] and have a low water vapor transmission rate than the transpiration rates of fresh products [159]. This condition generates two problems with the packaging of fresh mushrooms. On the one hand, an anaerobic atmosphere is generated in the containers that are used in MAP. Beit-Halachmy and Mannheim found that active MAP using PVC film is not suitable for mushrooms (*Agaricus bisporus*) due to their high respiratory rate [157]. In turn, an O_2_ concentration lower than 2% could cause anaerobic respiration and the potential growth of anaerobic pathogens [160]. On the other hand, the condensation of water inside the film is favored, which promotes decomposition, microbial growth, and browning of the product surface [161,162].

##### Microperforated Food Packaging and Moisture Regulators

To overcome these limitations, the use of microperforated films has been recommended to provide a greater range of permeabilities to O_2_ and CO_2_ and prevent anaerobic respiration of the packaged product [163]. Simon et al. [164] studied the use of perforated and nonperforated PVC films and two types of polypropylene microperforated films in MAP for the preservation of fresh sliced mushrooms. They found that the polypropylene film with the lowest permeability was the best at maintaining the quality attributes of the sliced mushrooms. However, when the storage temperature increases, more perforations are needed in the cellophane films that surround the trays to obtain the optimal conditions in MAP of sliced mushrooms [154]. In another study, the effect of perforation (0, 20, 40 and 60) mediated MAP on the shelf life of paddy straw mushroom (*Volvariella volvacea*) untreated and treated with CaCl_2_ was studied. The perforation mediated MAP effectively increased the shelf life of paddy straw mushroom to six days as compared to 1–2 days under normal condition. In general terms, pretreatment with CaCl_2_ (0.5%) and the use of a low-density polyethylene packages with 40 perforations (6.8 × 10^−4^%) generated the least weight loss, best maintenance of firmness, content of total phenols, antioxidant capacity, total protein content and the lowest total bacterial counts [146].

Because the microperforated films used for the packaging of fresh products do not allow the diffusion of enough water vapor into the packaged environment, the condensation of water vapor in the packaging is facilitated [73]. Moisture absorbers such as sorbitol [157] and silica gel [165] have been used to control the relative humidity inside the container and avoid the negative effects of water condensation on the conservation of mushrooms (*Agaricus and Pleurotus*). Recently, the development of moisture regulating trays by incorporating NaCl (6, 12, and 18%) into the packaging material has been researched to study its effect on the quality of fresh mushrooms [44,73,166]. The results indicated that the shelf life of the mushrooms was significantly increased, by six days, compared to that under conventional packaging at 5 °C. On the other hand, the water vapor condensation behavior indicated that after six days of storage at 7 °C and 85% relative humidity, the trays absorbed only 4.1 g of water vapor, which was not enough to prevent water condensation in the headspace of the container.

Although all these preservation methods have the ability to increase the shelf life of mushrooms, they also have their own drawbacks, including safety considerations, decreases in nutritional value, discoloration, textural changes, contamination by pathogens, bad flavors, high cost of capital, high energy consumption, and consumption unsuitable at an industrial scale [167,168]. For this reason, the combination of novel conservation techniques and conventional techniques could be a successful strategy to generate a synergy that allows them to more efficiently maintain the quality and extension of the postharvest shelf life of fresh mushrooms [57].

## 5. New Packaging Processes

In recent years, research on food packaging has increased considerably because consumers demand healthier and safer foods. This has forced the food industry to develop new food packaging strategies, including active packaging and smart packaging [169].

### 5.1. Active Packaging

Active packaging is an innovative technology used to extend the shelf life of packaged foods. It is based on the incorporation of antimicrobial agents, antioxidants, and carbon dioxide emitters/generators in the container [170]. Active packaging can progressively release active agents to the surrounding atmosphere or absorb compounds that deteriorate the food, such as oxygen and free radicals, extending the freshness of food products [54]. The incorporation of four active agents with antimicrobial and antioxidant properties (sodium metabisulfite combined with citric acid, green tea extract, cinnamon essential oil, and purple carrot extract) in a microperforated polypropylene container was studied to preserve fresh sliced mushrooms (*Agaricus bisporus*) [54]. The active containers containing sulfur dioxide and green tea extract at high concentrations managed to maintain the white color of the mushrooms for longer than the conventional container. The cinnamon essential oil and purple carrot extract did not show strong enough antioxidant properties to extend its shelf life [54]. In another study, the effect of the combined use of MAP and bilayer active packaging, formed by a layer of gelatin containing pomegranate peel powder on a polyethylene film, on the shelf life of oyster mushrooms (*Pleurotus ostreatus*), was studied [51] The results indicated that the controlled package atmosphere with a mean concentration of oxygen and with active bilayer packaging increased the shelf life to eleven days compared to three days in the control.

Because food products are complex systems, the parameters for active packaging must be specific to each product, so it is crucial to consider all the factors that may influence the outcome, such as the physical, chemical, and physiological properties of the food, the size of the container, and the storage conditions. The cost of implementation must correspond to the benefit added to the food product, legislative and regulatory issues must also be addressed, and wide acceptance by the consumer is required [171]. Some of the obstacles or limitations that can be identified in active packaging are finding an appropriate antioxidant that works in a comprehensive manner, is safe, and can be incorporated into the packaging material without any problems [54].

### 5.2. Intelligent Packaging

Intelligent packaging is one of the newest technologies in the field of food packaging. Although this technology is still under development and has not come to market, it has enormous potential to improve the safety, quality, and traceability of food products, as well as their convenience to consumers [172].

The concept of intelligent packaging has been defined in different ways by different researchers. According to Jang and Won [173], intelligent packaging is a packaging system that detects, communicates, and monitors the conditions of packaged foods to provide information on the quality, safety, and history of a product during transport and storage. To perform the monitoring of the packaged product, a series of sensors, indicators (temperature, freshness, leaks, and pH), and data carriers (barcode and radiofrequency identification labels) can be used [169]. These packaging systems can detect and control the necessary parameters to maintain the quality of the product [174]. Table 2 details different studies in which active and intelligent packaging has been tested on mushrooms.

Intelligent packaging is easy to use and offers advantages through the use of indicators and sensors but has some limitations [180]. It must be ensured that the system is compatible with the food to be monitored. Not all smart packaging can be used for all types of food [180,181].

Another aspect that still needs to be clarified is the recycling of intelligent packaging. The additional waste generated by the installation and production of intelligent packaging is actually contradictory to the objective of reducing food waste [180,182]. It should also be noted that it is not possible to rely 100% on intelligent packaging for optimal product quality, since misuse or failure of the systems cannot be ruled out [170]. Often, several factors are responsible for the loss of quality of a product, and the monitoring of a single parameter cannot provide a complete statement on the quality status of a product [182].

## 6. Sustainable Food Packaging

It is estimated that more than 30% of food produced deteriorates during transport and/or harvest [183]. Therefore, the use of a packaging that reduces and prevents the generation of food waste is required. Food packaging plays an important role in society, protecting food from potential damage and degradation while ensuring safety and hygiene and actively reducing food waste [184]. Plastics are the most commonly used materials to manufacture food packaging thanks to such advantages as low cost, low weight, versatility, flexibility, transparency, heat sealing, and good mechanical and barrier properties [185]. However, conventional packaging, especially those derived from petroleum, such as polyethylene, polypropylene, and polyethylene terephthalate, have a huge environmental impact because most of them are single-use containers that are used intensively and come from nonrenewable and nonbiodegradable sources [186] and in some cases are difficult to recycle [187]. In addition, current consumers are increasingly aware of and sensitive to environmental problems, which influences their behavior and consumption pattern.

These facts have forced the scientific sector to boost its research efforts and the food packaging industry to implement strategies to improve the sustainability of its packaging under the concept of a circular economy [188]. According to Peelman et al. [189], the sustainability of food packaging can be achieved at three levels: (1) at the level of raw materials, through the use of recycled materials and renewable resources to reduce the emission of CO_2_ and the use of fossil resources; (2) at the production level, through more energy efficient processes; and (3) at the waste management level, considering reuse, recycling, and biodegradation.

In this same sense, many countries have established various environmental laws and regulations to encourage the sustainable production of food packaging and thus help reduce their environmental impact. Some of the regulations seek to reduce or eliminate the use of single-use plastics, and other laws seek to promote recycling and waste management and establish extended producer responsibility. In general, these laws aim to reduce the generation of waste and promote its reuse, recycling, and other types of valuation of recyclable materials.

Normally, fresh mushrooms are packed in expanded polystyrene trays and coated with a PVC or polyethylene film. However, in some countries, such as France, the use of polystyrene will be eliminated before 2025 and PVC before 2022, according to what was declared in the National Pact on Plastic Packaging [190].

This scenario forces the scientific sector studying packaging and the industrial sector producing it to explore the development or use of new materials with smaller environmental impacts. One of the promising classes of materials for the manufacture of containers from renewable sources are biopolymers [191]. The great challenge that these materials face is that they must have physical and mechanical properties that can preserve food similarly to synthetic packaging.

## 7. Conclusions

This review highlights the importance of the chemical and nutritional composition of mushrooms and the nutraceutical and therapeutic benefits that some of their bioactive compounds have on the health of consumers. Different factors associated with the rapid degradation of the quality of mushrooms after harvest were reviewed, as were several traditional and emerging techniques for the preservation of fresh mushrooms. In general, most of these preservation techniques have shown a positive effect on the maintenance of the main quality parameters of mushrooms. Some of these techniques have some drawbacks related to potential toxicity, effects on taste, high cost, and energy consumption. Different studies have shown greater effectiveness when using a combination of emerging and traditional preservation techniques to extend the shelf life of fresh mushrooms. Considering the benefits and limitations of the revised traditional and emerging conservation methods, it is possible to highlight the potential that the combined use of cooling, UV irradiation and active edible coatings could have as pre-treatments to control the growth of microorganisms, enzymatic activity and promote the production of vitamin D_2_. At the same time, it would be advisable to consider the use of modified atmosphere packaging that incorporates a humidity regulation system, to maintain the quality and nutritional composition of the mushroom during the marketing chain. However, it is also necessary to evaluate the economic impact that the applications of these combined methods would have in commercial applications. Most studies focus on the effectiveness of preservation techniques at maintaining quality parameters such as color, texture, and flavor, which are associated with shelf life and the consumer’s purchase decision. However, there is little information on the effects of these preservation techniques on the protection of the main active and nutraceutical compounds, which are relevant to the health of consumers. It is also necessary to incorporate sustainability requirements in the development of packaging for the preservation of fresh mushrooms.

## Figures and Tables

**Table 1:** Different packaging techniques for postharvest conservation of mushrooms.

Materials	Film Material	Process Parameters	Results	References
Synthetic	Expanded polystyrene.	Polystyrene tray wrapped with PVC film. Storage temperature of fresh mushrooms (*Pleurotus ostreatus*): 5 ± 1 °C.	Increased shelf life to 12 days.	[145]
	Mediated perforation low-density polyethylene film.	Film thickness: 95 μm Perforation diameter: 0.45 mm. Number of perforations in container: 0, 20, 40, 60. Storage temperature of fresh mushrooms (*Volvariella volvacea*): 12 (±1) °C. Pretreatment: half of the samples were treated with 0.5% CaCl_2_.	The atmosphere mediated by the perforation increased the shelf life of the mushroom to 6 days. Mushroom firmness was better preserved with packages with 20 and 40 perforations. There were no significant differences in total protein content between the 20- and 40-perforationg groups, but total phenolic content, antioxidants, and bacterial count were best in the pretreated samples stored in containers with 40 perforations.	[146]
	Biaxially oriented polypropylene bags.	Pretreatments: immersion in different concentrations of 2,2′-(hydroxynitrosohydrazino)-bisethanamine (DETANO) for 10 min Storage temperature of fresh mushrooms (*Agaricus bisporus*): 4 °C.	Treatment with 1 mM DETANOmaintained a high level of firmness and delayed browning and cap opening. DETANO in combination with modified atmosphere extended storage life up to 12 days.	[147]
	Low-density polyethylene Film thickness: 0.04 mm.	Storage temperature of fresh mushrooms (*Agaricus bisporus*): 4 °C. Relative humidity: 85%.	Elevated CO_2_ reduced the browning of fungi and increased total phenolic content and activity and total antioxidant activity, extending the shelf life of the button mushroom.	[56]
Biodegradable	Prepackaging trays from agricultural waste fibers.	Prepackaging trays made from rice-industry waste for fresh mushrooms (*Agaricus bisporus*) at a temperature of 4 °C and 25 °C.	The weight losses of the mushrooms remained below 5%, and the Hunter L values were above 76 at 4 °C, while the mushrooms packed in trays of rice pulp showed a greater lightness than the ones packed in polystyrene trays stored at 25 °C.	[148]
	Films of a mixture of poly (lactic acid) (PLA) and poly(ε-caprolactone) (PCL) with different cinnamaldehyde concentrations.	Storage temperature of fresh mushrooms (*Agaricus bisporus*): 4 ± 1 °C and relative humidity: 85%.	The greatest weight loss of the mushrooms packed with PLA and PCL was 3.08% at the end of storage. The level of CO_2_ inside the PLA and PCL films with cinnamaldehyde was lower than that of the PLA and PCL films without cinnamaldehyde, but the level of O_2_ in these two types of films was similar.	[149]

**Table 2:** Applications of active and intelligent packaging in mushrooms.

Elements	Function/Results	References
Sulfur dioxide, green tea extract, cinnamon essential oil, purple carrot extract. These active agents were incorporated on filter paper as an active label and on polyethylene terephthalate film.	Active agents incorporated to extend the shelf life of fresh mushrooms. Active packaging exhibited antioxidant properties without being in direct contact with the mushrooms while maintaining the white color.	[54]
Macroperforated polyethylene terephthalate trays covered with cinnamon essential oil and cinnamaldehyde active papers.	Active paper had elements that preserved the mushrooms against oxidation, inhibiting tyrosinase to extend their shelf life.	[175]
Film with the addition of active agents such as grafted chitosan and gallic acid and polyethylene films.	Film used as a new active packaging material for the conservation of *Agaricus bisporus*, promoting the maintenance of its postharvest quality. There was a lower respiration rate and a lower degree of browning but a higher antioxidant status than those packed with chitosan and polyethylene films.	[176]
Chitosan/zein (tocopherol) films as packaging for mushrooms.	Active film used to extend the shelf life of *Agaricus bisporus*, which improve the deterioration of the quality of the mushrooms, reducing weight loss, maintaining firmness, and decreasing browning.	[177]
Packaging paper prepared with 1-methylcyclopropene and potassium permanganate.	Functional paper used for the elimination of ethylene and to increase the storage time of the mushroom *Agaricus bisporus*; delayed the softening, browning, and weight loss of mushrooms during the storage period. It had an acceptable quality, with weight loss of 4.66%, firmness of 0.79 kgf, lightness of 86.11, and browning index of 36.18 after 6 days of storage.	[178]
Polytextile, thermoregulatory material coated with microencapsulated paraffin melamine powder.	Intelligent packaging used for temperature regulation (5 °C) of mushrooms. Quality parameters were kept within the acceptable limits.	[179]

## Data Availability

Not applicable.
